# The neurogenesis of P1 and N1: A concurrent EEG/LFP study

**DOI:** 10.1016/j.neuroimage.2016.09.034

**Published:** 2017-02-01

**Authors:** Michael Bruyns-Haylett, Jingjing Luo, Aneurin J. Kennerley, Sam Harris, Luke Boorman, Elizabeth Milne, Nicolas Vautrelle, Yurie Hayashi, Benjamin J. Whalley, Myles Jones, Jason Berwick, Jorge Riera, Ying Zheng

**Affiliations:** a School of Systems Engineering, Whiteknights, University of Reading, Reading RG6 7AY, United Kingdom; b Department of Psychology, University of Sheffield, Sheffield S10 2TP, United Kingdom; c Department of Biomedical Engineering, Florida International University, Miami, FL 33174, United States of America

**Keywords:** P1, N1, event related potentials (ERPs), Local field potentials (LFPs), somatosensory evoked potentials (SEP), bicuculline, excitation, inhibition, whisker barrel cortex, rat, electroencephalogram (EEG), somatosensory cortex

## Abstract

It is generally recognised that event related potentials (ERPs) of electroencephalogram (EEG) primarily reflect summed post-synaptic activity of the local pyramidal neural population(s). However, it is still not understood how the positive and negative deflections (e.g. P1, N1 etc) observed in ERP recordings are related to the underlying excitatory and inhibitory post-synaptic activity. We investigated the neurogenesis of P1 and N1 in ERPs by pharmacologically manipulating inhibitory post-synaptic activity in the somatosensory cortex of rodent, and concurrently recording EEG and local field potentials (LFPs). We found that the P1 wave in the ERP and LFP of the supragranular layers is determined solely by the excitatory post-synaptic activity of the local pyramidal neural population, as is the initial segment of the N1 wave across cortical depth. The later part of the N1 wave was modulated by inhibitory post-synaptic activity, with its peak and the pulse width increasing as inhibition was reduced. These findings suggest that the temporal delay of inhibition with respect to excitation observed in intracellular recordings is also reflected in extracellular field potentials (FPs), resulting in a temporal window during which only excitatory post-synaptic activity and leak channel activity are recorded in the ERP and evoked LFP time series. Based on these findings, we provide clarification on the interpretation of P1 and N1 in terms of the excitatory and inhibitory post-synaptic activities of the local pyramidal neural population(s).

## Highlights

• Concurrent recording of somatosensory evoked potentials and field potentials in rat.
• P1 solely reflects excitatory postsynaptic activity, and indexes cortical excitation.
• Initial slope of N1 in deeper cortical layers solely reflects excitation.
• N1 width and peak amplitude modulated by inhibitory postsynaptic activity.

## 1 Introduction

Increasing evidence suggests that excitatory and inhibitory synaptic inputs co-tune in individual cortical neurons, with inhibition lagging excitation by several milliseconds (Anderson et al., 2000, Atallah and Scanziani, 2009, Higley and Contreras, 2006, Okun and Lampl, 2008, Turrigiano, 2008, Wehr and Zador, 2003, Xue et al., 2014, Zhang et al., 2003). This phenomenon is generally referred to as balanced excitation and inhibition, and has been observed in both evoked and spontaneous neural activity. However, it is unclear how this balanced synaptic activity at cellular level is reflected in the temporal dynamics of extracellular field potential (FP) recordings, such as local field potentials (LFPs) and electroencephalogram (EEG). When considering the origin of these signals, it is generally accepted that they primarily reflect the weighted sum of synchronised excitatory and inhibitory synaptic activities of local pyramidal neural populations (Buzsáki et al., 2012, Einevoll et al., 2013, Logothetis, 2003, Mitzdorf, 1985). This implies that the temporal dynamics of FP recordings are shaped by, among other factors, the ratio of excitatory to inhibitory synaptic strength, the distribution of the temporal lag of inhibition with respect to excitation of pyramidal neurons within the population, and the degree of synchronised neural activity responding to synaptic inputs.

Interpretation of FP recordings in terms of synaptic excitation and inhibition is difficult because different types of synaptic activity from pyramidal neurons can elicit identical FP temporal profiles (Logothetis and Wandell, 2004), e.g. positive outward and negative inward ionic currents within a close spatial vicinity cannot be distinguished from one another in FP recordings. Despite these challenges, various studies have set out to understand the neurogenesis of LFP and EEG through innovative experimental paradigms and analysis techniques.

Previous *in vivo* electrophysiological studies demonstrated that the major afferent input to the neocortex originates from the thalamus and terminates mainly in layer IV, but also in layers V and VI (Armstrong-James et al., 1992, Bannister, 2005, Bruno and Sakmann, 2006, Constantinople and Bruno, 2013, Oberlaender et al., 2012, Roy et al., 2011). Furthermore, current source density (CSD) analysis of LFP recordings have linked P1 to the earliest sinks in the granular and infragranular layers (Castro-Alamancos and Oldford, 2002, Di et al., 1990, Jellema et al., 2004, Mitzdorf, 1985). However, fluorescent labelling analysis also showed that a large number of thalamocortical neurons converge on layer I in the neocortex (Rubio-Garrido et al., 2009), raising the possibility that inhibitory synaptic activity in the supragranular layers may influence the temporal profile of the P1 wave. Using topographic analysis, Di & Barth (1991) found that P1 in somatosensory evoked potentials arose from a more distributed neural population than the N1 wave, and suggested that P1 may not be the exclusive result of specific thalamocortical afferents. It has also been hypothesised that P1 reflects inhibition during early access to a complex knowledge system (Freunberger et al., 2008, Klimesch, 2011, Klimesch et al., 2007), although no electrophysiological evidence has yet been presented to support this hypothesis.

The aforementioned studies illustrate the diversity of competing interpretations regarding the origin of P1 in evoked LFP/EEG recordings. The aim of this study is to investigate the neurogenesis of P1 and N1 using concurrent EEG and LFP in the somatosensory cortex of rodent. To do so, we pharmacologically altered the balance between neural excitation and inhibition by micro-injecting sub-convulsive concentrations (10 µM) of bicuculline methiodide (BMI), a competitive gamma-aminobutyric acid (GABA_A_) receptor antagonist, into the somatosensory barrel cortex (S1BF) of anaesthetised rats to isolate glutamatergic receptor function (Johnston, 2013, Jones and Barth, 2002, Krishek et al., 1996, Ueno et al., 1997). LFPs and ERPs induced by electrical whisker stimulation were concurrently recorded from S1BF before, during and after drug injection via a 16-channel fluidic laminar micro-electrode and an EEG spider electrode. The temporal dynamics of P1 and N1 were compared in terms of their slopes, peaks and latencies.

## 2 Materials and methods

### 2.1 Animal preparation and electrophysiology

Detailed surgical procedures were published in our previous work (Boorman et al., 2015, Bruyns-Haylett et al., 2013, Kennerley et al., 2012, Slack et al., 2016). Female Lister Hooded rats weighing between 200 and 260 g were anaesthetised via intraperitoneal injection of urethane (1.25 g/kg); supplementary doses of 0.1 ml were administered if required. The contralateral whisker pad of each animal was electrically stimulated with a rectangular current pulse stimulation of 0.3ms width and an intensity range between 0.8–1.6 mA. Such stimulation produced no reliable changes in the mean arterial blood pressure (MABP), CO_2_ partial pressure, or heart rate, thereby precluding changes in systemic physiology that may have affected evoked neural responses.

### 2.2 Experimental procedures

#### 2.2.1 Study 1: LFP - saline/BMI injection

Animals (Study 1a, saline: n=8; Study 1b, BMI injection: n=12) were placed in a stereotaxic frame (World Precision Instruments), and the skull surface was exposed. A dental drill was used to thin an area (~1×1 cm^2^) of the skull overlying the somatosensory cortex to translucency to locate the SIBF using optical imaging. Details of the localisation procedure have been described previously (Boorman et al., 2015, Slack et al., 2016).

After the barrel cortex was located, a small hole (diameter: ∼600 μm) was drilled through the thinned skull directly over the area of identified cortex, and the dura was pierced with a 27-gauge needle. A stereotaxic arm was used to insert a 16-channel multi-laminar recording micro-electrode fitted with an injection port (100 μm spacing, area of each site 177 μm^2^, injection site located at the tip of the electrode near channel 16; NeuroNexus Technologies) perpendicular to the cortical surface to a depth of 1600 μm into the S1BF. The fluidic electrode was coupled to a Tucker David Technologies (TDT) preamplifier (PZ5) connected to a data acquisition unit (RZ2, TDT) via fiber optic cable. All neural data were sampled at 24.41 kHz with 16-bit resolution, and collected using OpenEX software (TDT), which also handled trigger timings and data storage.

#### 2.2.2 Study 2: concurrent EEG/LFP - BMI injection

**Fig. 1:**
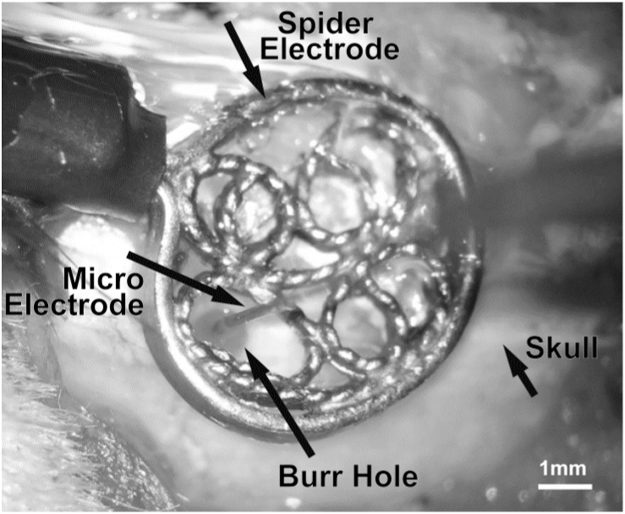
Set-up for concurrent LFP and EEG recordings. A burr hole was created over S1BF. A spider electrode was placed above the skull and secured using collodion adhesive. Non-conductive mineral oil was placed within the burr hole, and conductive EEG gel was placed between spider electrode and the skull around the burr hole.

Animals (n=9) were placed in a stereotaxic frame and the skull surface was exposed. It was considered important to preserve the integrity of skull conductivity for concurrent EEG and LFP recordings, so a small burr hole (1~2 mm in diameter) was drilled into the skull above the S1BF for electrode insertion. The location of the burr hole was guided by stereotaxic coordinates: 6mm laterally and 2.5mm posterior with respect to bregma (Paxinos Gand Watson, 1986). After drilling the burr hole, an EEG spider electrode (GVB-geliMED, Germany. Diameter: 6mm) was placed over the hole, with care taken to allow space through which the multi-laminar electrode could be inserted (Fig. 1). The spider electrode, which was made from Ag/AgCl, was of a flat reusable design, was then fixed to the skull with collodion adhesive. As per Study 1, a fluidic electrode was inserted perpendicular to the cortical surface to a depth of 1600 μm in the S1BF. Once the fluidic electrode was in place, the burr hole was filled with non-conductive mineral oil to prevent current leakage (Riera et al., 2012). The spider electrode was connected to the PZ5 via a passive signal splitter (S-BOX, TDT) for low impedance signals. EEG gel was applied to the spider electrode over the area of interest, with care taken to prevent contact with the multi-laminar electrode, and impedance was kept below 5 k Ω (Teplan, 2002). Again, as with Study 1, all neural data were sampled at 24.41 kHz with 16-bit resolution, and collected using OpenEX software (TDT), which also handled trigger timings and data storage.

### 2.3 Pharmacological manipulation of neural inhibition

The choice of BMI (Sigma-Aldrich, UK) concentration to reduce GABA_A_ modulated inhibition within the cortex was decided prior to the main studies, and guided by two criteria. First, the concentration should induce a marked effect on the temporal dynamics of the evoked LFP. Second, injection of BMI should not provoke a large number of epileptiform spikes in the LFP response. Five different concentrations of BMI (dissolved in 0.9% saline) were tested at a rate of 0.2 μl/min for 2 min: 200 µM, 100 µM, 50 µM, 25 µM and 10 µM. At all concentrations tested above 10 µM, pronounced epileptiform activity could be observed, whereas little to no epileptiform activity was present at a 10 µM concentration. Furthermore, micro-injection of BMI at 0.2 μl/min for 2 min at this concentration achieved relatively marked, reproducible and reversible drug effects (Schwartzkroin and Prince, 1980). The evoked LFP returned to its baseline response approximately 20 min post BMI injection. This concentration and injection rate were therefore used for all intervention experiments, and the interval between consecutive drug interventions was kept beyond 20 min.

### 2.4 Pre-processing of electrophysiological data

Before averaging, the ERP and evoked LFP trial data were pre-processed as follows: (i) The stimulus artifact was removed. (ii) Each trial was zero-meaned by subtracting the mean value of the neural signal 200 ms prior to stimulus onset. (iii) Data were low pass filtered below 800 Hz using a 4th order Butterworth IIR type filter in both directions to avoid introducing any temporal shift in the data. This frequency is slightly higher than is traditionally used (up to ~500 hz) to investigate LFP activity (Einevoll et al., 2013), as a lower cut-off frequency had the tendency to distort the temporal dynamics of P1. (iv) The multi-laminar data was aligned across animals. For each animal the inverse Current Source Density (spline iCSD, source radius R=0.5 mm) analysis (Pettersen et al., 2006) with a Gaussian filter (λ=50 µm) was applied to the LFP data under the control condition to locate the layer IV sink (Mitzdorf, 1985), which was given by the largest negative peak occurring at a cortical depth below the pial surface. We tested a range of source radius from 0.5 mm to 2 mm, and found that it had little effect on the locations of the sources and sinks (see Supplementary Fig. S1), although it had a small effect on the amplitude of the sources and sinks. The CSD data were then aligned according to their sink locations across animals. The common sink was located in layer IV, ~600 µm below the pial surface and the aligned LFP data were obtained by linear interpolation, with the first channel set to a cortical depth of 100 µm.

After BMI injection, the evoked LFP data occasionally exhibited spiking activity post-BMI injection, therefore trials were excluded from analysis based on the following criteria:

I. An epileptiform spike was detected within a 1 s window prior to stimulation. Such a spike usually leads to significant attenuation or abolition of the subsequent stimulus evoked LFP response.
II. The standard deviation of the data 200 ms prior to stimulation was greater than 5 times the standard deviation of baseline prior to BMI injection.

Throughout this paper, we use channel 2 from the realigned LFP to reflect supragranular layers activity, channel 8 to reflect granular layer activity, and channel 15 to reflect infragranular layers activity in the barrel cortex.

### 2.5 Parameter calculation

The following parameters were extracted from individual trials of neural responses. Although typically the domain of EEG nomenclature, we have used the terms P1 and N1 to refer to the first positive and negative deflections in both evoked LFP and EEG responses.

*P1 Peak Amplitude*. P1 was only observed in ERPs and the supragranular layers of the evoked LFP.

*N1 Peak Amplitude, Full-Width at Half Maximum (FWHM)* and *Peak Latency*. These were extracted from ERPs and LFPs across all cortical layers.

*N1 Initial slope*. This is measured as the slope from 2–25% of the N1 peak amplitude. This parameter was only extracted from the evoked LFP in the granular layer where P1 is absent.

### 2.6 Statistical analysis

All statistical comparisons were made between pre-injection (control) and post-injection (BMI/saline) conditions. All parameters were normalised with respect to the control condition as follows: first, the mean of each parameter (e.g. P1 amplitude) under the control condition across animals was calculated, then each parameter was normalised with respect to this mean by dividing it under both conditions for all animals. Two-tailed Student's paired t tests were used for all comparisons unless otherwise stated. Significant differences between normalised parameters pre- and post-injection were tested at 5% level of significance. Analyses were performed using the Matlab^TM^ statistics toolbox. Finally, differences in normalised parameters are presented as mean±standard error (SE) in Tables.

### 2.7 Experimental design

#### 2.7.1 Study 1: LFP - saline/BMI injection

This study investigated changes in the temporal dynamics of evoked LFP recordings that occurred when reducing the inhibitory post-synaptic activity of the local neural population via BMI intervention. To ensure that any change observed in the LFP pre- and post-intervention was due solely to BMI, we included a saline study and injected saline at the same volume and rate as conducted in the drug study. In Study 1, concurrent cerebral blood flow (CBF) was also collected via a laser Doppler flowmeter (data not used in this paper), therefore the inter-stimulus-interval (ISI) was chosen to be 25 s to allow CBF to return to baseline within each trial. Each experiment contained at least 4 runs, and each run contained 55 trials of single pulse stimulation (1.6 mA) delivered to the contralateral whisker pad. The first 5 trials (125 s) of each run were control trials, and demonstrated typical laminar temporal profiles (Einevoll et al., 2007, Hewson-Stoate et al., 2005, Higley and Contreras, 2007, Martindale et al., 2003). Immediately upon cessation of trial 5, a programmable syringe pump (Aladdin AL-1000, WPI) was triggered (using the TDT system) to deliver BMI into the S1BF. As the micro-injection lasted for 2 min, trials 6–10 fell within the period during BMI injection. Trials 11 onwards were referred to as post-injection trials over which the effect of BMI on the temporal profiles of evoked LFP could be observed until eventually returning back to baseline. For each run, data were averaged into epochs of 5 consecutive trials, then averaged across 4 runs. This resulted in 11 averaged epoch responses across all animals, with each epoch containing 20 trials. The average LFP response of the first epoch (pre-injection) was referred to as the control response. The second epoch contained all LFP response during BMI injection, while the third and subsequent epochs were post-injection responses. This method of averaging resulted in a temporal resolution (125 s window) adequate enough to observe distinct changes in the temporal profile of the evoked LFP at different stages post-BMI injection.

#### 2.7.2 Study 2: concurrent EEG/LFP - BMI injection

The primary objective of this study was to investigate the relationship between temporal dynamics of the ERPs and the LFPs across cortical layers, and whether the ERP temporal dynamics were similarly altered by reducing the level of local synaptic inhibition via BMI injection. No hemodynamic data were collected for this study, thus allowing the ISI to be reduced to 5 s. In order to collect data comparable with that from Study 1, we used 1 run from each experiment, and each run contained 300 trials of single pulse stimulation (1.2 mA) delivered to the contralateral whisker pad. The first 25 trials (125 s) of each run were control trials against which results were compared. BMI was injected between trials 26–50, and trials 51 through to 300 were post-drug trials during which the initial growth and then gradual cessation of the effect of BMI on neural activity was observed. Data were averaged into epochs of 25 trials each (ISI=5 s), thus occupying the same 125 s duration as in Study 1. Again, as in Study 1, epoch 1 represents the control response, epoch 2 the response during BMI injection, and epoch 3 onwards the responses post-injection. For some subjects, we conducted further runs with different stimulus strength ranging from 0.8 mA to 1.6 mA to test the impact on FPs by varying the degree of afferent input to the cortex.

In the analysis presented below, we did not include neural response data collected during and immediately after BMI injection (epoch 2 and epoch 3 respectively), as they displayed high variance due to the injection process. For statistical comparison, epoch 1 data was used as the pre-injection response, and epoch 4 data as the post-injection response.

## 3 Results

### 3.1 Effect of saline injection (n=8, 1.6 mA)

**Fig. 2:**
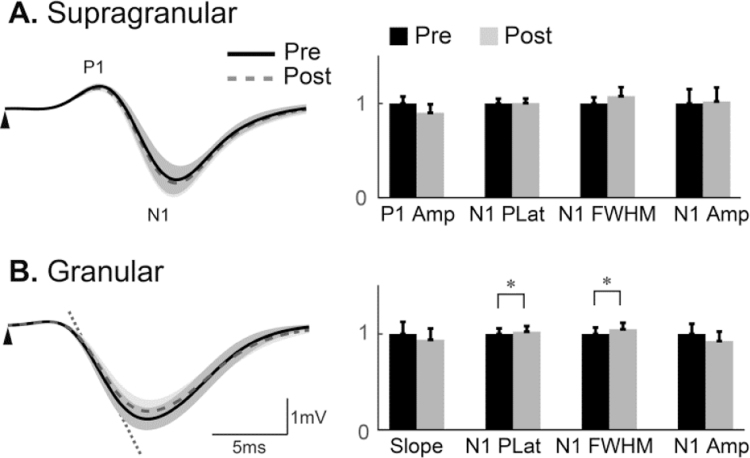
Comparison of evoked LFP between pre- (control) and post-saline injection (saline). Mean evoked LFP responses from the supragranular layers (**A*****,*** left panel) and granular layer (**B**, left panel). Saline responses (grey broken) almost overlapped control responses (black solid). Shadows indicate the standard error of control and saline responses across subjects. The triangle indicates stimulus onset. ‘P1’ and ‘N1’ illustrate the first positive and negative peaks of the supragranular LFP, whereas only N1 exists in the granular LFP. The initial slope of the granular LFP is illustrated on the left panel of **B**. Bar plots (right panels) show parameter comparisons between control and saline conditions normalised to the mean of the control condition across animals, and error bars indicate standard error. Parameters were compared using the two-tailed Student's paired *t*-test (* p<0.05). Amp: amplitude; FWHM: full width at half maximum; Plat: peak latency.

For the saline study, the evoked LFP time series in the supragranular layers pre- and post-injection largely overlap, as shown in Fig. 2A (left panel). Indeed, paired *t*-tests conducted pre- and post- saline injection showed no significant difference across all parameters (right panel of Fig. 2A, and Table 1). In contrast, the evoked LFP responses in the granular layer pre- and post-injection, although similar, show that N1 is slightly different pre- and post-injection (Fig. 2B: left panel). Parameter comparisons (Fig. 2B: right panel, and Table 1) show no significant difference between pre- and post-injection for N1 initial slope and peak amplitude, but show a marginal but significant difference in N1 FWHM and peak latency at the 5%, but not 1%, level of significance.

Our results suggest that saline injection (into layer VI of the cortex) has no significant effect on the evoked LFP response in the supragranular layer, but has a marginal yet significant effect on the pulse width and peak latency of the negative deflection in evoked LFP from the granular layer. These two parameters were significantly increased but the percentage increases were small, with the mean increase in N1 FWHM ~5%, and the mean increase in N1 peak latency ~2%. By contrast, the effect of BMI injection on these parameters was much larger (see below), with the mean increase in N1 FWHM exceeding 194%, and the mean increase in N1 peak latency exceeding 20%. Thus it was considered prudent to assume that the response after BMI injection (results presented below) was due to BMI rather than saline.

It is also worthwhile noting that our LFP amplitude across the cortical depth ranges between 1–4 mV, with LFP amplitude in the supragranular layer typically around 1.5 mV, becoming larger in the granular layer to be approximately 3.5 mV. These amplitudes are in-line with the published literature (Boorman et al., 2015, Devor et al., 2005, Higley and Contreras, 2007).

### 3.2 A change in inhibition did not alter the evoked LFP during the initial period of the response (n=12, 1.6 mA)

**Fig. 3:**
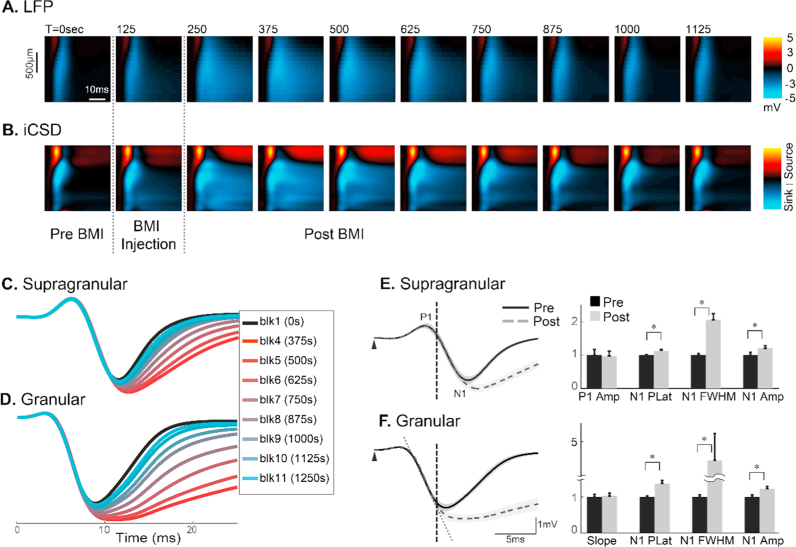
BMI Effect on the laminar LFP and CSD. **(A)** Progression of BMI effect on the laminar profile of evoked LFP. **(B)** Corresponding CSD calculated using the inverse Current Source Density (spline iCSD) analysis (see methodology); sources are indicated by red and yellow, whereas sinks are blue. For **A** and **B**: x-axis: time from the stimulus onset to 40 ms; y-axis, cortical depth 100 μm–1600 μm (top to bottom) from the pia mater. Each subplot shows an aligned average (n=12) of evoked LFP (**A**) or CSD (**B**) within 125 s period, with the starting time of the epoch period indicated at the top left of each subplot. The 10 images spanned 1250 s (~21 min), and contained pre-, during, and post-injection phases of BMI (bottom of **B**). **(C,D).** Progression of BMI effect on the temporal dynamics of the supragranular LFP (**C**) and granular LFP (**D**). Each curve represents an evoked LFP within an epoch averaged across animals. Data before BMI injection was labelled ‘blk 1’ (black). Post-injection, data were plotted from epoch 4 (red) through to epoch 11 (light blue). **(E,F).** Comparison of evoked LFP under pre- and post-BMI conditions. Mean LFP pre- (black solid) and post- (grey broken) BMI conditions are superimposed for the supragranular (**E,** left panel) and granular (**F**, left panel) layers. Shadows indicate the standard error of responses across subjects. The initial period post stimulus (<8 ms) is indicated in **E** and **F** by a vertical broken line. For symbols and abbreviation, refer to Fig. 2 legend. (For interpretation of the references to color in this figure legend, the reader is referred to the web version of this article.)

BMI was injected into the layer VI of the barrel cortex via fluidic micro-electrode. The progression of the effect of BMI injection on the stimulus evoked LFP across the six layers of the barrel cortex is shown in Fig. 3A, while the effect on current sources and sinks is shown in Fig. 3B. Each image displays the data averaged per epoch, e.g. the first image is the average neural response of epoch 1 across all cortical layers pre-BMI injection, the second is the epoch 2 data during injection etc. It can be seen that the effect of BMI on the LFP broadened the negative deflection (N1) across the entire depth of the cortex. Similarly, the current sink in the granular layer increased in duration.

To observe the progression of the effect of BMI injection on neural responses over time, we superimposed the evoked LFP from the different epochs in the supragranular and granular layers, shown in Fig. 3C&D respectively. It can be observed that the dynamics of the evoked LFPs within the first ~8 ms post-stimulus, encompassing the P1 wave and the initial downslope of the N1 wave, were unaffected by BMI-injection, whilst the peak amplitude, latency and pulse width of N1 all increased post-BMI injection across the cortical layers. From Fig. 3C&D, the BMI effect can be seen to be more prominent in the epoch 4 response (bright red) compared with that in epoch 5 and later. The evoked LFP responses pre- and post-BMI in the supragranular (Fig. 3E) and the granular (Fig. 3F) layers were plotted and compared using paired *t*-tests, shown in Table 2, which confirmed a statistically unaltered P1 in the supragranular LFP, and a statistically unchanged N1 initial slope in the granular LFP response (right panels of Fig. 3E&F, and Table 2). This suggests that the initial period (~8 ms) of the evoked LFP responses across the barrel cortex is related to excitatory post-synaptic activities of local neural populations.

By contrast, the later part of the N1 wave was significantly changed by BMI injection. From the left panels of Fig. 3E&F, N1 can be observed to have a larger amplitude, a longer peak latency, and a broadened pulse width post-BMI injection. These observations were confirmed statistically (Table 2).

### 3.3 A change in inhibition did not alter P1 in ERP: concurrent EEG/LFP (n=9, 1.2 mA)

The preceding findings inspired a study to determine whether the P1 and N1 components of the ERP would be affected by BMI injection using the same experimental paradigm.

The major difference between the evoked LFP and ERP appears to be that the latter records activity from a larger spatial extent, and could be an integration of synaptic neural population activity from further cortical regions (Nunez and Srinivasan, 2006). By collecting concurrent EEG/LFP, the relationship between the ERP and the evoked LFP across cortical layers could be explored. It is worth noting that a lower intensity of 1.2 mA was elected for this study, allowing us to test whether results from Study 1 would still hold under a different stimulus magnitude.

**Fig. 4:**
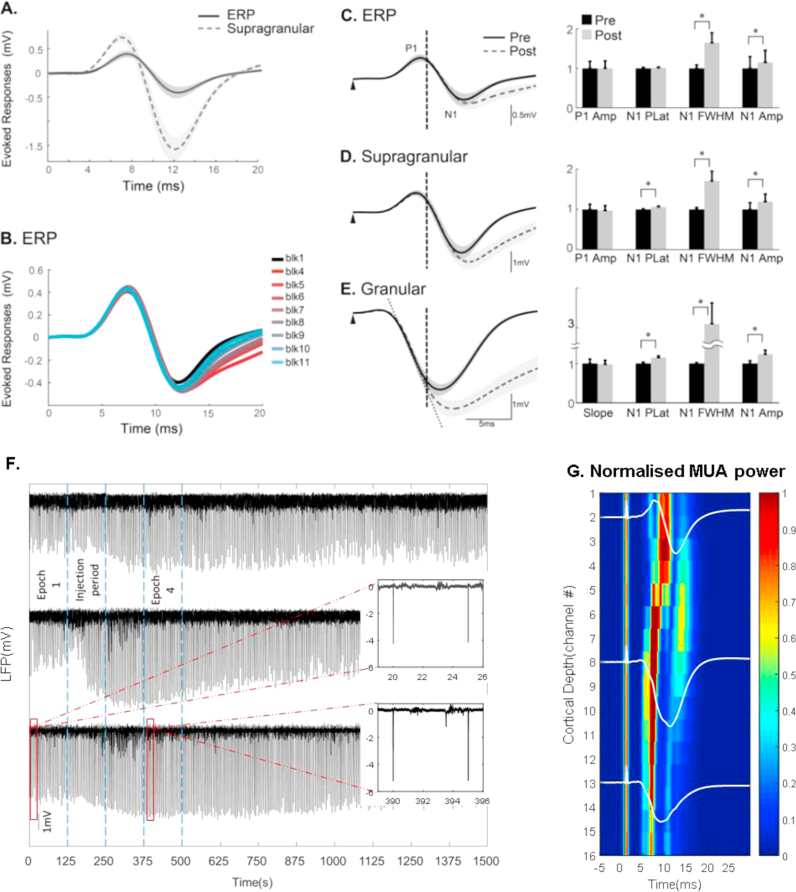
BMI effect on the concurrently recorded evoked LFP and ERP **(A)** Mean ERP (solid) and supragranular LFP (broken) from concurrent recordings are superimposed, with the shadow indicating standard error across subjects. **(B)** Progression of BMI effect on temporal dynamics of the ERP. Each curve represents an evoked LFP within an epoch averaged across animals. Data before BMI injection was labelled ‘blk 1’ (black). Post-injection, data were plotted from epoch 4 (red) through to epoch 11 (light blue). **(C,D,E)** Mean ERP from skull EEG (**C,** left panel), supragranular LFP (**D,** left panel), and granular LFP (**E**, left panel) were concurrently recorded. LFPs in control condition (black solid) and the BMI condition (grey broken) were superimposed. The initial period post stimulus (<8 ms) is indicated in **C, D** and **E** by a vertical broken line. Shadows indicate standard error. For symbols and abbreviation, refer to Fig. 2 legend. **(F)** Examples of raw granular LFP data from 3 subjects spanning 25 min including all trials pre, during and post-BMI injection. The insets show a zoomed-in period (7 s) of the LFP pre- and post-drug injection. **(G)** Normalised MUA (obtained via wavelet coefficients with centre frequencies 400:2000 Hz) from the control period meaned across all animals. Superimposed over the MUA are normalised plots of the LFP taken from the supragranular, granular and infragranular layers. (For interpretation of the references to color in this figure legend, the reader is referred to the web version of this article.)

The average ERP and supragranular LFP responses across animals during the control condition are shown in Fig. 4A, and demonstrate that the ERP was slightly delayed in time and its amplitude was about one third of that of the evoked LFP in the supragranular layers. The progression of the BMI effect on the temporal characteristics of ERP is illustrated in Fig. 4B. It shows that, similar to Study 1, the P1 wave and the initial slope of the N1 wave remained unchanged regardless of BMI intervention, while the later part of the N1 wave was modulated by BMI injection.

To demonstrate this more clearly, the ERP and the evoked LFPs pre- vs post-BMI are plotted in the left panels of Fig. 4 (C, D & E). Statistical analysis was performed to compare the temporal dynamics of the ERP with LFP under the control and the drug conditions, shown in the right panels. Table 3 lists the results of the statistical comparisons for the relevant parameters.

No significant difference was found in P1 amplitude of the ERP and the supragranular LFP responses, as well as in the initial slope of N1 in the granular layer. However the later part of the N1 wave was significantly modulated by BMI injection in ERP and LFP responses across the cortex, reflected in the peak amplitude, peak latency and FWHM of N1, with one exception that the peak latency of N1 in ERP did not significantly change.

We further tested if BMI significantly increased the baseline spiking activity of local cortical neurons at the selected dosage by calculating the multi-unit activity (MUA) of the extracellular FPs across cortical layers prior to the onset of stimulation (−1s to 0 s). For each trial the power spectral density (PSD) of the selected FP time course was calculated in Matlab^TM^ (using FFT with a Hanning window) and MUA was obtained by averaging the PSD over the frequency range 500–3000 Hz (Boorman et al., 2010). Paired *t*-tests were conducted across all 16 channels to compare the baseline MUA during the control and the drug period. No significant difference was found for any channels, with the p-value in the range [0.186 0.842]. We conclude that at the dosage of BMI used, the local neural population activity was minimally affected at the resting state. Fig. 4F displays the raw LFP time series (granular layer, 300 trials over 25 min.) from 3 subjects to provide an overview of the effect of the BMI injection. It shows that BMI may induce some spiking activity during and immediately after injection. But this activity subsided while the evoked LFP amplitude was still elevated post injection, including the test epoch 4. The number of epileptiform spikes between evoked responses was minimal, and typically only a single isolated spike was observed during epoch 4, as shown in the insets. The MUA activity of the resting LFP was otherwise unaltered.

To obtain insight into the cortical dynamics responsible for the P1 and N1 waves, we computed the time series of MUA across the cortical depth using the continuous wavelet transform (mother wavelet: biorthogonal 1.5) in Matlab^TM^. The mean modulus of the wavelet coefficients with centre-frequencies 400-2000 Hz was collected for each animal from the control period, meaned across all animals, and then normalised to the maximum value. Fig. 4G displays MUA across all channels from 5 ms pre- to 30 ms post-stimulus, with the evoked supragranular, granular and infragranular LFPs superimposed to better contextualise the temporal dynamics of the MUA. It shows the earliest MUA activity occurring in granular and infragranular layers, with MUA activity in the supragranular layers starting ~3 ms later and coinciding with the downslope of the P1 deflection.

**Fig. 5:**
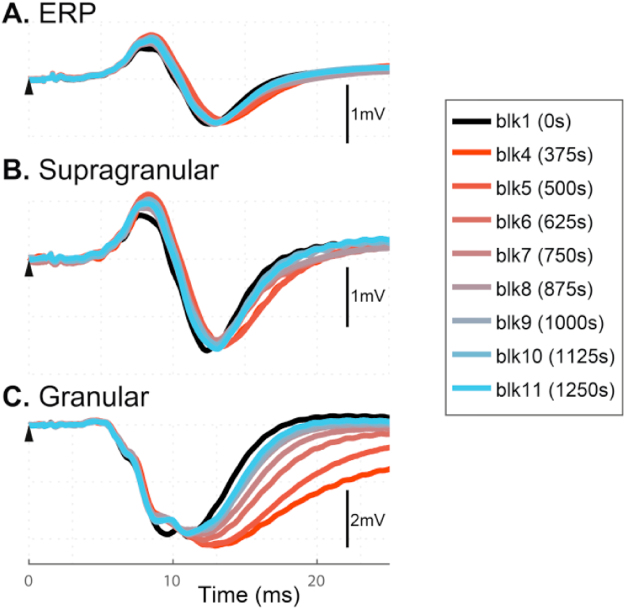
Example of disassociation between ERP and granular LFP (n=1). Temporal dynamics of the **(A)** ERP, **(B)** supragranular LFP and **(C)** granular LFP are displayed. In each subplot, a single curve represents an evoked LFP within an epoch averaged across animals. Data before BMI injection was labelled ‘blk 1’ (black). Post-injection, data were plotted from epoch 4 (red) through to epoch 11 (light blue). In this case, BMI injection produced a large effect on the temporal dynamics of the granular LFP, but little effect on the supragranular LFP and ERP. (For interpretation of the references to color in this figure legend, the reader is referred to the web version of this article.)

In most subjects, BMI-injection (in the infragranular layers) induced observable changes in the ERP. However, for a minority of subjects the BMI effect in ERP seemed marginal. If this was due to unsuccessful BMI injection, then the BMI effect in the granular layer would also be marginal. However, when the data were plotted (Fig. 5, single subject), we found obvious and expected changes in the granular layer LFP due to BMI injection, but both the supragranular LFP and the ERP were not significantly affected. This suggests that the ERP did not observably reflect the changes in evoked LFP from deeper cortical layers, but instead appeared to mainly resemble the supragranular LFP.

### 3.4 Stimulus intensity altered P1 and N1 (n=4)

It has been shown that the neural coding of stimulus intensity is best accounted for by the firing rate evoked in afferent neurons located within the proximity of stimulation (Muniak et al., 2007, Ray et al., 2008), with increased firing rate as stimulus strength is increased. This has also been reflected in the increase in amplitude of the evoked LFP (N1) recordings in layer IV of rodent barrel cortex (Li et al., 2011). Furthermore, investigation into the relationship between stimulus intensity and the sensory evoked primary sink of the rat barrel cortex showed the relationship to be approximately sigmoidal (Jones et al., 2004). Both the amplitude and the initial slope of the CSD sink were increased as the stimulus intensity was increased.

**Fig. 6:**
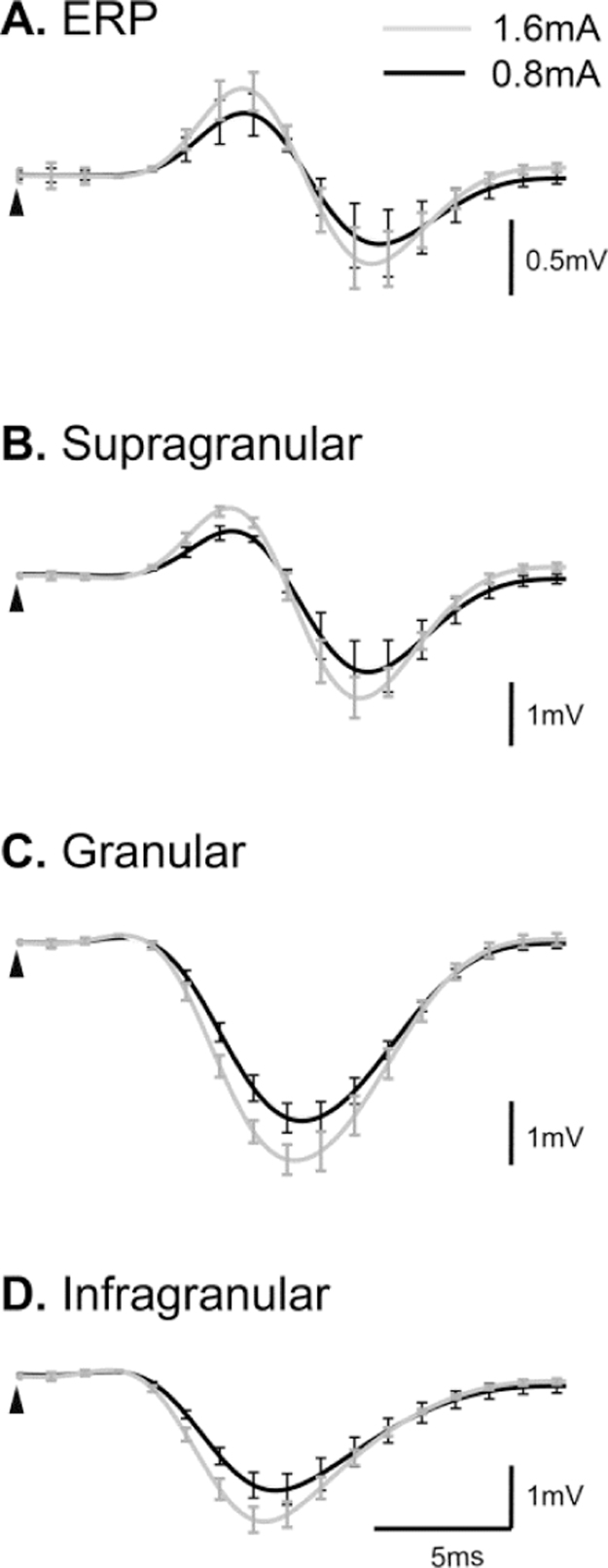
Comparison of FP recordings at different stimulus intensities (n=4). Temporal dynamics of the **(A)** ERP**, (B)** supragranular, **(C)** granular and **(D)** infragranular evoked LFP averaged across animals are displayed at stimulus intensities 0.8 mA (black) and 1.6 mA (grey).

We collected LFP data (n=4) for different stimulus strengths in order to contrast the stimulus-intensity-induced changes in extracellular FP with those induced by BMI-injection. Fig. 6 shows the FP recordings generated by 0.8 mA and 1.6 mA stimulus intensities respectively. As expected, an increase in stimulus intensity increased both P1 and N1. Furthermore, the gradient of the initial slope of the ERP and LFP across all cortical layers increased as stimulus intensity increased. This was in direct contrast to BMI-induced changes, in which no significant modification was observed in the early phase of sensory processing recorded in both EEG and LFP.

### 3.5 Power spectral analysis

**Fig. 7:**
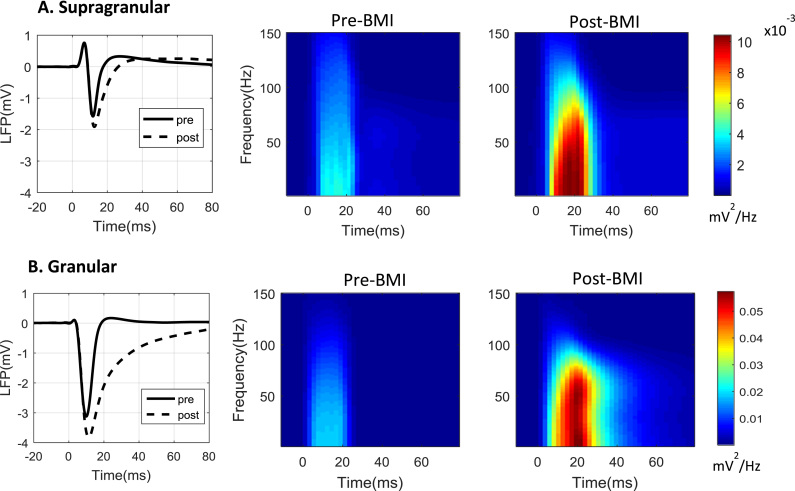
Power spectral analysis of the LFP pre- and post-BMI injection (n=9). (A) The evoked LFP responses of the supragranular layer pre- (solid line) and post- (broken line) BMI injection are shown in the left panel. Their spectrograms are shown in the middle and right panels respectively. (B) Same as (A) but for the granular layer.

To illustrate the effect of BMI-injection on the frequency characteristics of the evoked LFP we calculated spectrograms of the evoked LFP for the pre- and post-drug injection (epochs 1 and 4 respectively), and averaged across animals for study 2, as shown in Fig. 7. The power spectral density was calculated using the Matlab^TM^ function ‘pmtm’ which is based on the multitaper method (Thomson, 2000). The sliding temporal window length was chosen as 20 ms, giving a spectral resolution of 50 Hz. The power spectrum was then interpolated along the frequency axis by the ‘pmtm’ function to provide a smoother appearance. Note that the 20 ms window covered most of the evoked LFP deflections, rendering it impossible to characterise frequency content over the finer details of the LFP profile. However, any further reduction of this window length would lead to an undesirably poor frequency resolution.

From Fig. 7 it can be seen that for both the supragranular and granular layers, the power of the evoked LFP response within the frequency range 0–100 Hz post-BMI injection was much higher compared to that before the injection. However, any information regarding the initial slope and peak latency of the evoked LFP responses pre- and post-drug intervention was lost in these figures.

## 4 Discussion

Using the rat somatosensory barrel cortex as a model, we have presented the first direct experimental evidence that the P1 wave and the initial downslope of the N1 wave in the ERP and LFP recordings occur independently of inhibitory post-synaptic activity. This discussion will first address methodological issues related to our experimental procedures, and then provide interpretations of our findings in the context of earlier work.

### 4.1 Pharmacological intervention

We pharmacologically reduced synaptic inhibition by micro-injecting BMI at a sub-convulsive concentration into layer VI of the rat barrel cortex. At high concentrations or injection rate, the application of bicuculline is known to induce seizures (Ma et al., 2009, Yu et al., 2008). It also has side effects on calcium-dependent potassium channels in a concentration-dependent manner, blocking the current underlying the low-threshold spike burst afterhyperpolarisation at high concentrations (≥20 µM) (Debarbieux et al., 1998, Katzner et al., 2011, Kurt et al., 2006). To minimize these effects, we selected a lower concentration of 10 µM, an injection volume of 0.4 µl, and an injection rate of 0.2 µl/min. Furthermore, we calculated baseline MUA pre- and post-BMI injection across all cortical depths to determine whether the baseline firing rate changed under BMI. No significant change was observed 2 min after the cessation of BMI injection. This implies the difference between the evoked LFP under the control versus the drug condition was unlikely to be caused by different baseline conditions.

Our findings appear to contrast with those of Jones and Barth (2002), who investigated the effect of BMI on fast (>200 Hz) electrical oscillations in the rat somatosensory cortex using a laminar electrode array. Their BMI concentration was also 10 µM, but soaked into squares of filter paper and then applied to the surface of the cortex surrounding the electrode. FP recordings were taken while the filter paper was in position. They found that BMI not only modified the characteristics of the fast oscillations, but also induced a more rapid onset of P1 in the top channel, with increased P1 amplitude and pulse width. This was in disagreement with our findings which did not show significant changes in P1 after BMI injection. It is likely that this difference is related to the amount of BMI used in the two studies. We injected 0.4 µl BMI per experiment, whereas Jones and Barth (2002) used a considerably larger volume when soaking the filter paper. Our post-drug neural data was recorded ~2 min after the cessation of BMI injection, whereas the post-BMI neural data shown in Jones and Barth (2002) were collected while the BMI-soaked paper was placed surrounding the laminar electrode array. It is possible that changes observed in the P1 dynamics post-BMI were partly due to significantly elevated baseline neural activity, however, the baseline MUA was not presented in their study.

We also tested BMI injection in cortical layer IV to investigate whether our findings were affected by the location of the injection site. This was done by raising the fluidic microelectrode by ~0.7 mm stereotaxically. We observed no qualitative differences in the evoked LFP responses pre- and post-BMI injection between these two injection sites (supplementary Fig. S2). This suggests that at our chosen dosage of BMI injection, the drug diffused effectively from the infragranular up to the granular and supragranular layers in most cases within the timeframe of 2 min after the cessation of drug injection, and our findings were not qualitatively influenced by the site of drug injection within the granular/infragranular layers.

### 4.2 Concurrent EEG/LFP set-up

A single spider electrode (6 mm diameter) was placed atop the rat's skull directly above the somatosensory cortex to allow the concurrent recording of surface EEG along with intracortical LFPs via a laminar microelectrode. This set-up is not dissimilar to previous concurrent EEG/LFP studies conducted in primates (Musall et al., 2014). Our objective was to investigate whether the temporal window of excitation observed in sensory evoked LFPs could also be observed in ERP time courses. In human studies and for complex cognitive tasks, it is important to identify the spatial origin of the signals of interest (e.g. ERP and brain rhythms), as well as to separate them from measurement noise and movement artifacts. To achieve this, multichannel EEG accompanied by effective source separation and localisation algorithms are required (Makeig et al., 2004, Makeig and Onton, 2009). However, as the spatial location of interest (S1BF) had already been well-established in the presented paradigm, source localisation was not required and a single EEG probe was deemed sufficient for purpose.

It is worthwhile noting that ERPs and evoked LFPs primarily record excitatory and inhibitory post-synaptic activities of pyramidal neurons, hence no inference can be made from these recordings regarding the excitatory and inhibitory post-synaptic activity of interneurons. It is well known that, in addition to pyramidal neurons, inhibitory interneurons in the barrel cortex also receive thalamic afferents after whisker stimulation, and their conductance will change accordingly, but these changes are not reflected in ERPs and LFPs due to the morphology of interneurons (Logothetis, 2002). Therefore, in FP recordings the delay of inhibition with respect to excitation solely refers to the post-synaptic activity of local pyramidal neural populations.

#### 4.2.1 P1 is unaltered by changes in inhibitory synaptic activity

As P1 is unaltered by BMI injection, this suggests that the neural origin of P1 in extracellular FP recordings is related purely to changes in the excitatory post-synaptic activity of pyramidal neurons, and is not influenced by inhibitory synaptic activity. We argue that the early increase and the subsequent decrease of the P1 wave likely reflect different synaptic events. The onset of the P1 wave (~4 ms after stimulation) coincides with that of the N1 wave in deeper cortical layers. As blocking GABA_A_ channels with BMI did not alter the temporal profile of P1, and the MUA in the supragranular layers stayed roughly at baseline prior to the P1 peak (Fig. 4G), the rise of P1 can only be due to thalamocortical afferents arriving at the pyramidal neurons in granular and infragranular layers, which induce return currents that flow into the extracellular space through active and passive channels located on the ascending dendrites of these neurons in the supragranular layers. This is in agreement with previous findings using CSD and topographic analysis (Castro-Alamancos and Oldford, 2002, Mitzdorf, 1985, Ohl et al., 2000). However, the downslope of P1 cannot be due to passive leak currents as these would result in a continuous increase in FP in the supragranular layers. Instead we suggest there may be several possible sources for the downslope of P1. One may be the slightly delayed arrival of thalamic afferent to pyramidal neurons in supragranular layers (De Kock et al., 2007, Oberlaender et al., 2012), although direct thalamic projection in supragranular layers are rare (Bureau et al., 2006). Another may be the arrival of feedforward afferent from layer IV pyramidal neurons (Armstrong-James et al., 1992, Constantinople and Bruno, 2013, Feldmeyer et al., 2002). Additionally, the early part of N1 waves in the deeper cortical layers, themselves generated by thalamocortical afferents (see below), may propagate through the cortical tissue and contribute to the downslope of P1. In EEG literature, the rising and falling slopes of the P1 wave in the visual evoked potential were found to originate from different anatomical regions of the visual cortex (Di Russo et al., 2002). Our results show support for this finding, as they suggest the likely components underlying the early and late phases of the P1 wave also originate from different sources, albeit importantly linked solely to excitatory synaptic activity.

It is well known that P1 is strongly influenced by stimulus parameters such as the stimulus strength (Luck, 2005), and we illustrated this with our data (Fig. 6) by comparing results from 1.6 mA versus 0.8 mA stimulus strengths. In this case, an increase in P1 amplitude as well as the gradient of the rising and falling slopes of the P1 wave can be observed when stimulus strength is increased. Based on our finding that P1 is independent of synaptic inhibition, we suggest that these increases in the P1 wave are exclusively due to an increase in thalamocortical afferent activity projecting to deep cortical layers, yielding an increase in the leak currents in the supragranular layers, followed by subsequent increase in thalamocortical/intracortical afferents to layers II/III.

Finally, it should be noted that the study presented here did not include short-term synaptic depression due to repeated stimulation (Castro-Alamancos, 2004, Heiss et al., 2008, Higley and Contreras, 2006), surround suppression in sensory integration (Carandini et al., 2002, Freeman et al., 2002, Gentet et al., 2010, Haider et al., 2010, Isaacson and Scanziani, 2011, Ozeki et al., 2009, Sachdev et al., 2012, Zhang et al., 2014), or neural modulation during multimodal sensory processing (Iurilli et al., 2012, Lee et al., 2013, Olcese et al., 2013). Our conclusion that the P1 wave is dependent solely on excitatory synaptic activity is valid only for evoked LFPs and ERPs with temporal dynamics not influenced by preceding stimuli. The mechanisms underlying neural modulation observed in evoked LFP and ERP when multiple stimuli from the same or different sensory modalities are involved are still hotly debated and intensely researched.

#### 4.2.2 Parameters of N1 reflect properties of synaptic excitation and inhibition

Our experimental results showed the initial downslope for the surface N1 wave (in ERP and supragranular LFP) was unaltered by BMI injection, suggesting that the initial phase of the surface N1 has the same neurogenesis as the later part of P1 and is solely related to excitatory post-synaptic activity. However the later phase of the surface N1 wave was significantly modulated by inhibitory post-synaptic activity (Fig. 3, Fig. 4). The more inhibition was suppressed, the larger and broader the N1 amplitude and pulse width respectively.

Similarly, inhibition has no influence during the early phase of the N1 wave in the granular layer (Fig. 3, Fig. 4), but modulates the later phase in terms of the N1 peak, peak latency and pulse width. The neurogenesis of the downslope of the N1 wave is well-accepted to be the arrival of thalamic afferent to layer IV, the main input layer of the neocortex (Feldmeyer, 2012, Oberlaender et al., 2012). Importantly, this implies that the initial slope of N1 in deeper cortical layer LFP recordings can be used to index changes in excitatory post-synaptic activity of local pyramidal neurons. The initial slope of evoked LFP in deeper cortical layers has been observed to be influenced by a number of conditions, such as activity-induced changes in brain temperature, the environment or drugs (Kublik, 2004, Moser et al., 1993), stimulus-related changes (e.g. stimulus strength), as well as changes in brain state (wakefulness or sleep) (Castro-Alamancos and Oldford, 2002, Martin et al., 2006, Vyazovskiy et al., 2008). Based on our findings presented here, these slope changes are not related to inhibitory synaptic activity, instead they may reflect changes in the strength of thalamic afferents arriving at the thalamocortical synapses, and/or the excitatory synaptic efficacy in the neocortex. This conclusion is in line with electrophysiological evidence (Castro-Alamancos and Oldford, 2002, Vyazovskiy et al., 2008). It is also highly likely that corresponding but delayed changes in inhibitory synaptic activity occur to balance excitation and alter the subsequence temporal profile of the LFP time course, however this inhibitory synaptic activity cannot be observed in extracellular FP recordings in isolation unless adequate mathematical models are used (Zheng et al., 2012).

The amplitude of the N1 peak can be influenced by both synaptic excitation and inhibition. The larger the evoked excitatory post-synaptic activity of the local pyramidal neural population the steeper the initial slope of the N1 wave, which in turn is likely to generate a higher N1 peak. On the other hand, for a given magnitude of evoked excitatory post-synaptic activity, weaker evoked inhibitory post-synaptic activity also has the tendency to increase the N1 peak, as our results indicated. Thus the amplitude of the N1 peak alone is not an indicator of synaptic excitation.

One of the consequences of a reduction in local inhibition is an inevitable increase in recurrent neural activity, which will be reflected by an increase in the N1 pulse width (Reinhold et al., 2015). Further factors influencing the pulse width of the N1 wave include sensory evoked slower excitatory and inhibitory post-synaptic activities mediated by N-methyl-d-aspartate (NMDA) and GABA_B_ receptors respectively (Benarroch, 2012, Lester et al., 1990). Further research into the effect of these on the temporal dynamics of ERPs and evoked LFPs will be needed to enhance our understanding of extracellular FP recordings.

#### 4.2.3 ERP is a close analogue of evoked LFP in supragranular layer

A strong similarity between the temporal profiles of ERP and supragranular LFP was observed, although by comparison the ERP demonstrated a slight temporal delay and was significantly attenuated. Not surprisingly, the BMI injection affected ERP temporal profile. We sometimes found that the ERP did not observably reflect the BMI induced changes in evoked LFP from deeper cortical layers (Fig. 5), but instead appeared to mainly resemble the supragranular LFP. This is important as it suggests that ERP measured from a single skull location, although recording activity from a greater lateral spatial spread than evoked LFP, does not appear to record activity deeper than the supragranular layer. It has been shown through a biophysical modelling approach that LFPs decay strongly with distance even in a purely resistive medium (Łęski et al., 2013, Lindén et al., 2011). Our data corroborated such findings and demonstrated that large field potentials generated in deeper cortical layers may be greatly attenuated by neural tissue, and only have a weak influence on EEG recordings. This highlights the need for further research into the neurogenesis and modelling of EEG signals (Acar et al., 2016, Lucka et al., 2012).

#### 4.2.4 Time vs frequency domain analysis of neural signals

Neural data can be analysed with a wide range of data analysis tools. The choice is dependent on the nature of the data obtained and the information of interest to be extracted. For example, measures of spontaneous brain activity recorded with microelectrodes or EEG often demonstrate oscillations at distinct frequencies which are related to different brain states ((Buzsaki, 2006; Csicsvari et al., 1999; Klausberger et al., 2003; Klimesch, 2013). Although the mechanisms underlying these signals are not understood, frequency domain analysis is more appropriate at identifying the frequency bands and the strength of these oscillations. Furthermore, many external stimuli, particularly those related to visual stimulation paradigms, are periodic by design. In such studies, frequency domain analysis is the logical choice to identify active regions of the brain by isolating the steady state evoked potential in a narrow frequency band centred on the stimulus frequency (Nunez and Srinivasan, 2006).

On the other hand, ERPs are generally time-locked to stimuli, and therefore can be aligned to the stimulus onset and averaged across trials (assuming normally distributed random noise) to increase the signal-to-noise ratio. Often the parameters of interest in experiments utilising ERPs are the peak amplitude and latency of individual deflections in the ERP profile. After averaging, these parameters can often be observed in the temporal profile of the neural recordings, which can be easily extracted, and subsequently used as markers of performance during cognitive tasks or in assisting diagnosis of certain neurological diseases (Luck, 2005, Sur and Sinha, 2009). These temporal characteristics are best analysed using time domain rather than frequency domain techniques.

In this paper we investigated the neurogenesis of P1 and N1 observed in SEPs by pharmacological interventions and comparing the temporal dynamics of P1 and N1 before and after BMI intervention. We used time domain analysis of the SEP, which allowed us to visualise and statistically compare the temporal dynamics (initial slope, peak latency and the FWHM) pre- and post-drug. Comparing these parameters then enabled us to isolate a period during which only excitation could be observed in the population recording. Solely conducting a frequency domain analysis of the SEP would not have revealed this finding.

#### 4.2.5 Mathematical models of EEG and LFP

Increasingly, mathematical models of extracellular FPs are developed to enhance our understanding of brain function (Bojak et al., 2010, Coombes, 2010, Deco et al., 2008, Einevoll et al., 2013, Jansen et al., 1993, Lindén et al., 2010, Pinotsis and Friston, 2011, Sotero et al., 2010, Valdes et al., 1999, Zheng et al., 2012). Some of these models are designed to understand brain connectivity and rhythms using resting state FP recordings, others are focused on modelling evoked LFP and their neurogenesis. Our findings may provide a vital constraint to neural population models by highlighting the importance of including the appropriate inhibitory delays in both feedforward and feedback loops, as well as incorporating the co-tuning property of excitatory and inhibitory events in the modelled pyramidal neural populations.

#### 4.2.6 Application to human EEG studies

Although our findings come from the rat somatosensory cortex, recent intracellular studies have demonstrated that inhibition lags excitation across all primary sensory cortices (visual: (Xue et al., 2014, Yang et al., 2013), auditory: (Sun et al., 2010, Wehr and Zador, 2003, Zhang et al., 2003), olfactory: (Fu et al., 2014), and somatosensory: (Gabernet et al., 2005; Gentet et al., 2010; Heiss et al., 2008; Higley and Contreras, 2006; Okun and Lampl, 2008; Wilent and Contreras, 2005)). We therefore suggest that the temporal window of excitation may be a common property across all sensory evoked FP recordings. Furthermore, sequential laminar activations in neocortical circuitry are well preserved across species (Arezzo et al., 1979, Arthurs et al., 2000, Di et al., 1990, Ebersole and Chatt, 1984, Kulics and Cauller, 1986, Raizada and Grossberg, 2003), implying the presence of fundamental cytoarchitectural features common to the mammalian columnar neocortex.

Indeed, when focusing on somatosensory evoked potentials, they display robustly similar structures across primary cortices in rats, primates and humans, albeit at different latencies (Allison and Hume, 1981, Kulics and Cauller, 1986). Many factors affect these timings, including the distance the neural signal travels (rat: from whisker pad to S1BF; human: from fingertip to somatosensory cortex), and the dynamics of cortical synaptic channels.

A number of challenges exist when translating our findings to human EEG studies. The first challenge is when navigating the different terminology used for evoked potentials across domains and species. Typically, the first positive deflection observed in SEP recordings from the primary rat somatosensory cortex post stimulation is termed P1, and the first negative deflection N1 (Di and Barth, 1991, Franceschini et al., 2008, Freeman and Sohmer, 1996, Jellema et al., 2004). However, the nomenclature used for describing human and macaque SEP ERPs either refer to the order in which they’re seen (e.g. P1a, P1b, N1) or the time (in ms) at which they are typically observed (e.g. P20, N30). In addition, as these components (e.g. N1) have often been named with a specific species in mind, they do not necessarily share the same neural source origins across species. Another challenge is when trying to infer specific generators solely by topographic analysis of surface recordings in human EEG studies. This is a difficult task due to the complex infolding of the human cerebral cortex and the resulting summation of volume conducted activity from several sources. The macaque model has often been employed to address this problem as it most closely resembles the human sensorimotor cortex and facilitates invasive recordings. Here, Arezzo et al. (1981) found the P12-N25 (referring to the time in ms at which they are observed) generated in anteromedial portion of the hand area of the somatosensory cortex in the macaque (median nerve stimulation at contralateral wrist) corresponded to the P25-N35 in human, and that the P10-N20 component overlying and anterior to the central sulcus corresponded to the P20-N20 component observed in human. Indeed, Allison et al. (1989) investigated anatomic generators of human SEPs (again, median nerve stimulation) in the 20-40ms interval with cortical surface and transcortical recordings during neurosurgery and from chronically implanted depth probe recordings in epileptic patients. They concluded that the P20-N30 and P25-N35 potentials were equivalent to the primary evoked response recorded from the somatosensory cortex of other mammals. These potentials would then be equivalent to the P1-N1 complex we observed in the rat somatosensory cortex (Freeman and Sohmer, 1996). Indeed, in spite of the differences in modality, they concluded that the P1-N1 complex observed in the rat somatosensory cortex in response to whisker stimulation was equivalent to that observed in response to forepaw stimulation, and as such regarded the whisker evoked SEP in rats as an ideal model for trigeminal SEP in man. Based on the results from the current study, this suggests that the P20/P25 component in human SEP might be viewed as indices of local cortical excitation, and that changes in the width of the N30/N35 component might be an indication of shifts in the balance between excitation and inhibition.

In conclusion, as our results show that both evoked LFP and EEG during early sensory processing are purely dependent on synchronised local excitatory synaptic events, our findings offer the opportunity for more accurate interpretations of LFP and EEG recordings, and provide an important constraint when establishing mathematical models of neural populations within the cerebral cortex. In addition, as changes in P1 could be used to indicate changes in synaptic excitation of the local cortical network, there is potential for this knowledge to grant further insight into pathological and normal brain function when interpreting extracellular recording techniques such as EEG.

## Figures and Tables

**Table 1:** Mean±can be seen to be more prominent inSE (p-value) of the differences in normalised parameters pre- and post-saline and the corresponding p values (*p<0.05) for saline study.

	Supragranular LFP	Granular LFP
P1 amp diff	−0.10±0.06 (0.14)	N/A
N1 init. slope diff	N/A	−0.06±0.03 (0.05)
N1 FWHM diff	0.08±0.04 (0.13)	0.05±0.02 (0.015)*
N1 amp diff	0.02±0.02 (0.40)	−0.07±0.03 (0.07)
N1 latency diff	0.004±0.003 (0.22)	0.02±0.01 (0.012)*

**Table 2:** Mean±SE (p-value) of the differences in normalised parameters pre- and post-BMI and the corresponding p values (*p<0.05) for BMI Study with LFP.

	Supragranular LFP	Granular LFP
P1 amp diff	−0.03±0.06 (0.66)	N/A
N1 init. slope diff	N/A	−0.03±0.03 (0.35)
N1 FWHM diff	1.06±0.19 (0.0003)*	3.49±0.75 (0.0007)*
N1 amp diff	0.21±0.04 (0.0002)*	0.23±0.02 (0.0000)*
N1 latency diff	0.13±0.03 (0.0007)*	0.38±0.08 (0.0007)*

**Table 3:** Mean±SE (p-value) of the differences in normalised parameters pre- and post-BMI and the corresponding p values (*p<0.05) for BMI study with EEG/LFP.

	ERP	Supragranular LFP	Granular LFP
P1 amp diff	0.01±0.04 (0.88)	−0.03±0.04 (0.59)	N/A
N1 init. slope diff	N/A	N/A	−0.02±0.04 (0.64)
N1 FWHM diff	0.65±0.20 (0.012)*	0.71±0.23 (0.014)*	1.76±0.55 (0.013)*
N1 amp diff	0.15±0.07 (0.05)*	0.19±0.07 (0.018)*	0.24±0.08 (0.016)*
N1 latency diff	0.02±0.03 (0.54)	0.07±0.02 (0.005)*	0.15±0.03 (0.0006)*
