# Supplementary material for: The neurogenesis of P1 and N1: A concurrent EEG/LFP study
Source: Neuroimage. 2017 Feb 1;146:575–88. doi: 10.1016/j.neuroimage.2016.09.034 (PMC5312787; doi:10.1016/j.neuroimage.2016.09.034)
Supplement: Supplementary Figure S1 — Supplementary material [file mmc1.docx]

Time(ms)

Cortical Depth (Channel #)

Sink Source

**Figure S1.** Effect of increasing source radius R.

The CSD images were calculated from a single subject neural recordings during the control condition. Spline iCSD was used with the source radius changing from 0.5mm to 2mmm in steps of 0.5mm.

Pre

Post

**Figure S2.** Comparison of evoked LFP between pre- (control) and post-BMI injection into cortical layer 4 (n=5).

Mean evoked LFP responses from the supragranular layers (top panel) and granular layer (bottom panel). LFPs in control condition (solid) and the BMI condition (broken) were superimposed. Shadows indicate the standard error across subjects.
